# Green Synthesis of Metal Nanoparticles with Borojó (*Borojoa patinoi*) Extracts and Their Application in As Removal in Water Matrix

**DOI:** 10.3390/nano14181526

**Published:** 2024-09-20

**Authors:** Erika Murgueitio Herrera, Gissela Jacome, Carina Stael, Geovanna Arroyo, Andrés Izquierdo, Alexis Debut, Patricio Delgado, Gemma Montalvo

**Affiliations:** 1 Centro de Nanociencia y Nanotecnología, Universidad de las Fuerzas Armadas ESPE, Av. Gral. Rumiñahui s/n, Sangolqui P.O. Box 171-5-231B, Ecuador; castael@espe.edu.ec (C.S.); gvarroyo@espe.edu.ec (G.A.); padelgado2@espe.edu.ec (P.D.); arizquierdo@espe.edu.ec (A.I.); apdebut@espe.edu.ec (A.D.); 2 Departamento de Ciencias de la Tierra y de la Construcción, Universidad de las Fuerzas Armadas ESPE, Av. Gral. Rumiñahui s/n, Sangolqui P.O. Box 171-5-231B, Ecuador; gejacome2@espe.edu.ec; 3 Departamento de Ciencias Exactas, Universidad de las Fuerzas Armadas ESPE, Av. Gral. Rumiñahui s/n, Sangolqui P.O. Box 171-5-231B, Ecuador; 4 Departamento de Química Analítica, Química Física e Ingeniería Química, Universidad de Alcalá, Ctra. Madrid-Barcelona km 33.6, 28871 Alcalá de Henares, Madrid, Spain; gemma.montalvo@uah.es; 5 Instituto Universitario de Investigación en Ciencias Policiales, Universidad de Alcalá, Libreros 27, 28801 Alcalá de Henares, Madrid, Spain

**Keywords:** arsenic, nanoparticles, borojó (*Borojoa patinoi*), Papallacta Lagoon

## Abstract

The predominant aim of the current research was to generate a proposal for the removal of arsenic, a highly toxic pollutant, encountered within the Papallacta Lagoon in Ecuador. The average concentrations of As yielded ranges between 18 to 652 μg/L, through the use of metallic nanoparticles. Sampling was performed in the lagoon with their respective geographic locations and “in situ” parameters. Nanoparticles of Mn_3_O_4_ NPs, Fe_3_O_4_ NPs, and CuO NPs were synthesized at a 0.5 M concentration, using the precipitation method, and borojó (*Borojoa patinoi*) extract was added as an anti-caking agent as well as antioxidant. The nanoparticles were characterized by visible spectrophotometry, scanning electron microscopy (SEM), transmission electron microscopy (TEM), X-ray diffraction (XRD), and Raman spectroscopy. After arsenic removal treatment using nanoparticles, a randomized experimental design of different concentrations (5 mg/L, 10 mg/L, 25 mg/L, 50 mg/L, 100 mg/L, and 150 mg/L) was applied at laboratory level. The average diameter of Fe_3_O_4_NPs ranged from 9 nm to 36 nm, Mn_3_O_4_ NPs were 15–20 nm, and CuO NPs ranged from 25 nm to 30 nm. Arsenic removal percentages using Fe_3_O_4_ NPs with a concentration of 150 mg/L was 87%; with Mn_3_O_4_ NPs, the removal was 70% and CuO NPs of about 63.5%. Finally, these nanoparticles could be used in a water treatment plant for the Papallacta Lagoon.

## 1. Introduction

Arsenic is a toxic and ubiquitous metalloid that is able to act as a geogenic contaminant. It is widely distributed in nature, present in the air, water, and soil. It can be released into the environment through various natural, agricultural, and industrial processes [1,2,3]. This element can be found in two states, organic as well as inorganic, while in its inorganic state, it is the more dangerous version. Arsenic exists in several chemical forms, each having a different mobility, which explains its toxicity. Hereby, As^3+^ (arsenite) and As^5+^ (arsenate) are the most stable and toxic forms of arsenic [4,5]. In humans, the main route of exposure to arsenic is through ingestion of food or water, while two further routes may be through inhalation of air and through the skin. Arsenic causes abnormalities such as a variety of cancers, abnormalities in fetal development, and genetic mutation, as it accumulates in biological organisms [5,6,7]. Drinking water is affected in its availability and safety due to geogenic contaminants such as arsenic in groundwater. There are millions of people who can suffer serious health problems and may even die, due to high concentrations of this metalloid that exceed recommended concentrations [8]. Numerous epidemiological and scientific studies have demonstrated that ingestion of arsenic through drinking water may be a causal factor in urogenital cancers, especially bladder cancers [9].

There are several reports of arsenic exposure from drinking water in Argentina, Chile, Mexico, El Salvador, Nicaragua, Peru, Bolivia, Spain, Thailand, and China. It should be noted that the World Health Organization (WHO) categorized arsenic as a chemical element harmful to public health, indicating 10 µg L^−1^ for drinking water and 100 µg/L for irrigation water [1]. The study of the Papallacta Lagoon is fundamental because it supplies one-sixth of the water to the population of Quito, Ecuador’s capital, and its inhabitants. Those are mostly rural people who depend on the water and food of this area, and are exposed to a real risk of contamination due to the daily consumption of water containing arsenic [10,11]. Lagoon ecosystems are aquatic environments of critical importance for their multifunctional role in maintaining biodiversity and sustaining coastal communities [12,13]. One of these projects was realized at the CENCINAT (Center for Nanoscience and Nanotechnology of the University of the Armed Forces ESPE, in Ecuador), which identified arsenic concentrations in the Papallacta Lagoon ranging between 390 and 670 μg/L. All of these concentrations are much higher than those recommended by the WHO [14].

There are several techniques to remove arsenic from contaminated water. However, their effectiveness and costs vary widely. Among the techniques used to remove arsenic from water are coagulation, filtration, membrane separation, and ion exchange, among others. Nanotechnology offers a process that could be economically viable and innovative, applying metallic nanoparticles [15,16,17,18].

Nanoparticles of the transition metal oxides of iron, manganese, and copper are rich in oxidation states, participating in reversible redox reactions, low cost, abundant on Earth, and harmless to the environment [19]. These oxides have been used in several studies for the removal of organic contaminants and even for the improvement of biodiesel using magnetite [20,21,22,23,24,25,26], while hausmannite and oxides of manganese at nano and micro levels have been used in detection [27,28] and water treatment process [29,30] for the elimination of organic and inorganic contaminants such as arsenic [31]. CuO Nps have been synthesized with a green procedure and used in the removal of dyes, namely methylene blue (MB) and methyl orange (MO), from contaminated waters through adsorption on CuO nanoparticles [32,33,34], photodegradation of tetracycline [35], for arsenic removal [36,37], and furthermore has been combined with magnetite also for arsenic removal [38].

Most nanoparticle synthesis methods described to date rely heavily on the use of organic solvents, toxic reducing agents, high temperatures, the use of organic solvents, toxic reducing agents, high temperature, and high pressure. Therefore, nowadays there is a need to develop sustainable ecological processes, the green chemistry, which lacks to use of toxic and dangerous materials. To support the non-use of toxic reagents in the preparation of nanomaterials and to be environmentally friendly, various plant materials have been used in green nanotechnology, this means that the reaction conditions, the reducing agent, the solvent medium, and the coating agent should be economically affordable and environmentally friendly reagents. Research has been realized where capulí, mortiño, and various fruits are used for the synthesis of nanoparticles [39,40,41], and the fruit extract has replaced the toxic reagents, constituting an ecological synthesis of nanoparticles.

In the present study, for the synthesis of nanoparticles, the borojó plant (*Borojoa patinoi*) has been used as an anti-caking agent for the nanoparticles and as an antioxidant due to its phenolic, flavonoid, and vitamin C properties [42]. It acts as a reducing agent in the formation of magnetite, as an anti-caking agent and stabilizer for hausmannite nanoparticles, and in the formation of copper oxide NPs due to the high concentration of ascorbic acid. The Fe_3_O_4_ NPs, Mn_3_O_4_NPs, and CuONPs nanoparticles together enhance the arsenic removal process, better than doing it individually. The synthesized NPs were characterized using different analytical instruments and discussed. Furthermore, the antioxidant efficacy of borojó was evaluated against 1,1-diphenyl-2-picrylhydrazyl (DPPHradical dot). Subsequently, the NPs were used to remove arsenic first at a laboratory level and could later be used in a water treatment plant for the Papallacta Lagoon.

## 2. Materials and Methods

### 2.1. Description of the Study Area

The Papallacta Lagoon is located in the Papallacta Parish belonging to the Quijos Canton of the Napo Province. It is situated on the Quito-Papallacta-Baeza highway, just two kilometers short reaching the town of the Papallacta Parish and two hours east of the city of Quito, in north-central Ecuador. Its geographic position is 00°22′10″ latitude (south) and 78°10′06″ longitude (west). This body of water is located between the Cayambe-Coca and Antisana National Parks, having a temperature of over 4 °C, while constantly warming up in the morning and cooling down at and during the night [43].

### 2.2. Sampling

**Figure 1:**
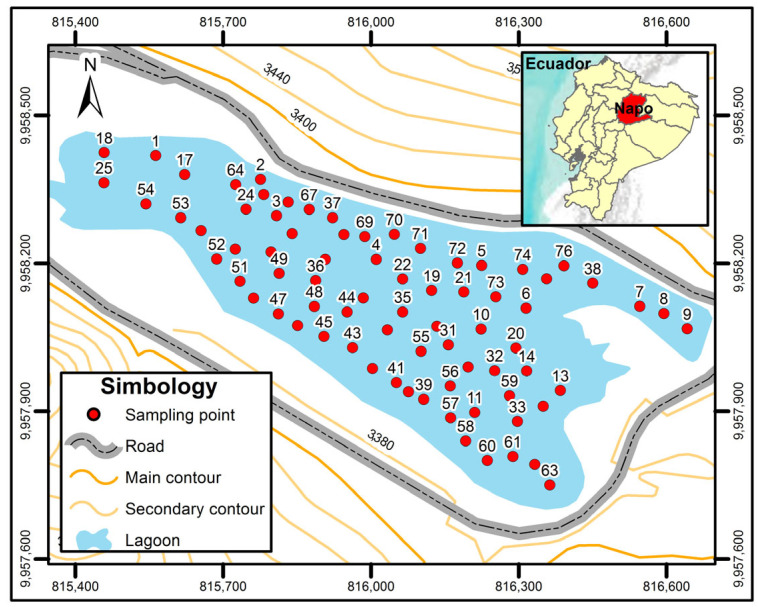
Location map of arsenic sampling points within the Papallacta Lagoon.

Seventy-five water samples were taken at the surface and subsurface in the Papallacta Lagoon, on three different dates, as in Ecuador there are only rainy and dry seasons (see Figure 1). The GPS Mobile Mapper Field Spectra 20 was used. The water samples were taken at a depth between 2 m and 15 m and with equivalent distances using a Kemmerer BTL 1.2 L SS [44]. These samples were collected according to the Ecuadorian technical standard for water quality, sampling, handling, and conservation of samples, and the random probability sampling technique was applied.

### 2.3. Chemicals

Reagents for water analysis were purchased from USA Hach Company P.O. Box 389 Loveland, Colorado. For the purification of the synthesis products and the preparation of the reaction solutions, ultrapure water from a Milli-Q system (Conductivity 0.28 µS/cm, Millipore, Direct Q, Darmstadt, Germany) was used, while the ethanol used was absolute grade, as with ACS (, ISO (Isopropyl Alcohol) 1728Batch 19.022.310.

### 2.4. Determination of Physical-Chemical Parameters

For the temperature (2550 method) and hydrogen potential (2550 method), as well as hydrogen potential (2550 method 4500-$H^{+}$ B) the HQ 30 d multiparameter equipment was used. Furthermore, for the sulfates (method 4500 S${O_{4}}^{=})$, nitrates (method 4500 N${O_{3}}^{-}$ B), and turbidity (nephelometric method 2130 B) we used a variety of methods as described in the Manual of Standard Methods for the Examination of Water and Wastewater (APHA, 1992), realized at a spectrophotometer with UV/Visible lamp (Hach model Specord S600, Analytic Jena, Thuringia, Germany) [45]. For the total determination of arsenic, a Perkin Elmer AAnalyst 800 atomic absorption spectrometer (EAA) from Shelton, CT, USA was used, applying its FIAS injection system and Perkin Elmer WinLab32 for AA; Version 7.4.1.0730 (2014). For this process, the 3500 B methodology for arsenic in section 3114 described in the Manual of Standard Methods for the Examination of Water and Wastewater (APHA, 1992) was used. A calibration curve is performed with annual technical maintenance.

### 2.5. The Extract Fruit/Plant Preparation

The borojó fruit was purchased in the local market of Sangolquí, located in the Cantón of Rumiñahui, within the province of Pichincha. Afterward, the sample was cleaned and disinfected. It was then dried at 40 °C in an oven and weighed as well as labeled [39,46]. For ethanolic extraction, pulp and peel were used, and a 1:1 solution was realized (extract mass: ethanol volume 70%). The solution was concentrated by orbital shaking for 72 h. The extract was separated from the ethanol with a rotary evaporator (Buchi 850, Flawil, Switzerland). The fruit extract was filtered several times through sieves and placed in Falcon tubes to be centrifuged for 15–20 min, filtered using membranes of pore size of 0.45 μm diameter and later of 0.22 μm, while the extract was stored at 10 °C. The accuracy and precision of the equipment were not achieved as it is limited to solvent extraction, which was not performed for the given measurements.

### 2.6. Characterization of Fruit

First, the antioxidant activity was evaluated by the DPPH method, using Genesys UV-Vis equipment, model 10uv, Software Thermo Scientifics VISIONlite Scan version 4.0 (2002–2009) from the USA (last maintenance performed in June 2023). The experiment was realized in triplicate. Subsequently, to determine the redox potential, the cyclic voltammetry method was applied (potentiostat AUTOLAB PGSTAT128N, 2014; low isolates), following the protocol according to the user manual. The results were analyzed using Minitab version21.3, year 2024. To determine if there was a significant difference in the averages, a multifactorial analysis of variance was applied, and significant differences between averages (*p* < 0.05) were identified using Fisher’s LSD test.

### 2.7. Synthesis of Nanoparticles of Fe_3_O_4_NPs, Mn_3_O_4_NPs, CuONPs

The NPs were prepared by the precipitation method and at room temperature of 20 °C, Fe_3_O_4_NPs were synthesized according to the existing protocol (Murgueitio’s et al., 2018) [18]. A 0.5 M solution of FeCl_3._7H_2_O (Batch SG34191112; Wodehouse Road, Mumbai 400005, India) was prepared. Borojum extract previously alkalinized with 1M sodium hydroxide was added to a pH between 9–10, with a volume ratio of (1:1). The pH was controlled with a multiparameter HQ equipment. Subsequently, they were dried on a heating plate at 80 °C. Hausmannite (Mn_3_O_4_) NPs were synthesized using a modified version of the Stähli procedure in alkali, based on the protocol in [46,47]. Two solutions of 0.5 M MnCl_2_, each prepared from MnCl_2._H_2_O (Sigma-Aldrich Laborchemikalien GmbH D-30926 Seelze lot 4265A, Steinheim, Germany), one at 20 °C and the other at <5 °C, were used. Alkalinized borojum solutions were added to each solution at pH 9–10. Subsequently, they were mixed in an oxygen line for 30 min and 20 °C. The solid obtained was centrifuged and washed several times with milliQ water.

The synthesis of the NPs-CuO was based on the existing protocol [46]. A 0.5 M solution of CuCl_2_ (CuCl_2._2H_2_O Sigma Aldrich, Saint Louis, MO, USA, CAS 10125-13-0) was sIG an aeration line was added for 30 min. The solid obtained was centrifuged and washed several times with milliQ water.

All the solids obtained were washed with abundant milliQ water, centrifuged (Hermle centrifuge, model Z206A, Gosheim, Germany), and filtered through filter paper and several filters of 0.45 μm and 0.22 μm, consecutively. They were subsequently stored in an airtight bottle for subsequent characterization, at room temperature (25 °C) and away from any exposure to light.

### 2.8. Materials and Equipment Used in the Characterization of NPs

The nanoparticles are characterized to determine their average size and morphology. In addition, data on absorption bands at specific wavelengths are provided. Particle size characterization was performed using TEM. The protocol consists of letting a drop of colloidal suspension of nanoparticles evaporate on a carbon-coated copper grid and then analyzed on a transmission electron microscope (model Fei Tecnai G2 Spirit Twin, Eindhoven, The Netherlands) operating at 80 kV [48]. Calibration involved the diffraction of the gold particles and the measurement of the distance between two gold nanoparticles (0.24 nm apart). The use of the pre-calibrated web for reference has been at https://www.agarscientific.com/tem/calibration-standards (accessed on 1 April 2020). The ImageJ-win64 software was used to analyze the size of the nanoparticles. In addition, a Genesys 10uv UV-Vis spectrophotometer equipped with the Thermo Scientifics VISIONlite Scan version 4.0 (2002–2009) Software (Waltham, MA, USA) was used, while maintenance is performed annually. The baseline is obtained using deionized water as reference and the method of analysis is performed according to the equipment manual Thermo Fisher Scientific (Waltham, MA, USA). Automatic self-calibration was performed to ensure optimal wavelength accuracy and reproducibility.

Finally, semi-quantitative analysis of the elemental composition of each sample was performed in the chamber of a TESCAN (Brno, Czech Republic) brand scanning electron microscope (SEM,), model MIRA 3, using the Bruker X-Flash 6|30 energy dispersive X-ray detector (Bruker, Berlin, Germany) with 123 eV resolution for MnKα [48]. Maintenance, alignment verification, and calibration are performed annually. Calibration was performed with the help of https://www.tedpella.com/calibration_html/SEM_Calibration.aspx (accessed on 1 April 2020) networks [48].

The Raman spectra were collected with a Thermo Scientific confocal DXR Raman spectrometer (Waltham, MA, USA), a 780 nm excitation laser was used for the iron oxides and copper oxide, for the manganese oxides a Raman Cora 5001 equipment was used, with a 785 nm excitation laser.

### 2.9. Arsenic Removal with Mn_3_O_4_ NPs, Fe_3_O_4_ NPs and CuO NPs

We worked with the arsenic concentrations of 5 mg/L, 10 mg/L, 25 mg/L, 50 mg/L, 100 mg/L, and 150 mg/L, in synthetic water in the laboratory, simulating the concentration of As total in the Papallacta Lagoon. Each arsenic dilution had three replicates to perform the arsenic removal tests, while to each was added a 1:1 ratio (*v*/*v*) of nanoparticles. They were then shaken in a Wise Shake SHO-2D digital orbital shaker for 24 h at 150 rpm. Subsequently, they were filtered several times to a 0.22 µm filter. Finally, the concentration of arsenic in the final solutions was determined. The analyses were realized individually for each type of NPs.

## 3. Results and Discussion

### 3.1. Physical-Chemical Analysis of Water Samples from the Papallacta Lagoon

**Figure 2:**
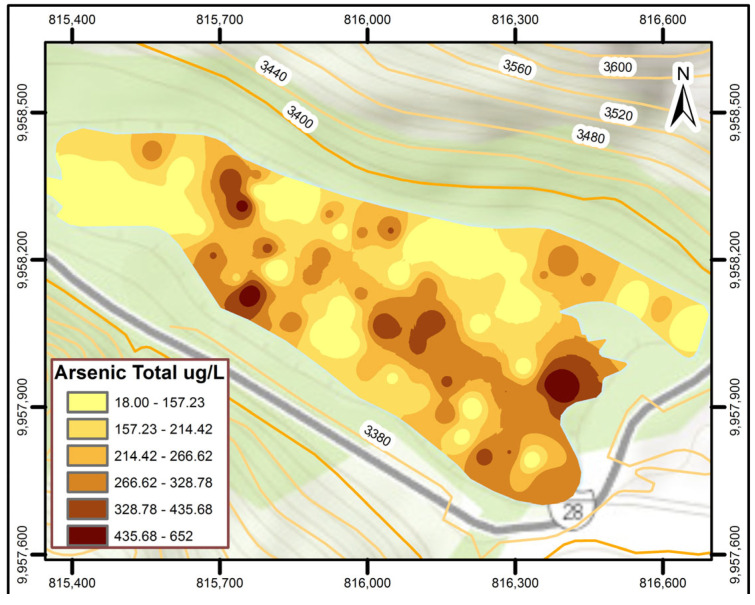
As total concentration in the Papallacta Lagoon.

Samples were taken during the rainy season, at a maximum depth of 15 m and a minimum depth of 2 m. The sampling points are indicated in Figure 1. The average water temperature was 12.76 °C ± 0.52, similar to that obtained in previous research [49] and the average pH was 7.43 ± 0.32. These pH values were similar to the pH values obtained previously [50], where pH values between 7 and 9 were obtained from the same lagoon. The average sulfate concentration was 42.5 mg/L, nitrates 2.2 mg/L, and turbidity 70.6 UTN (Nephelometric Turbidity Unit), which is within the established limits (Table 1 of Book VI of TULSMA Annex 1 [51]). The arsenic concentration data from the Papallacta lake obtained in this study range from 18 to 652 μg/L. Figure 2 demonstrates the distribution of As concentration in the lake, where the concentrations are lower than the concentrations previously obtained [52], where arsenic concentrations ranged from 677 to 1203 μg/L of As within a rainy season. This decrease in arsenic concentrations in the lagoon is attributed to the dilution conducted during the winter season in this body of water. During the winter season, the lagoon increases its volume from 8,000,000 m^3^ to a volume of approximately 13,500,000 m^3^.

### 3.2. Characterization of the Fruit Extract

The hydrogen donating capacity to DPPH radicals of 342.8 ± 31.0 µm Trolox equivalents/100 g fresh fruit, which is similar to that published by [53]. Compared to other fruits, the level is intermediate.

### 3.3. Characterization of Mn_3_O_4_ NPs, Fe_3_O_4_ NPs, and CuO NPs

**Figure 3:**
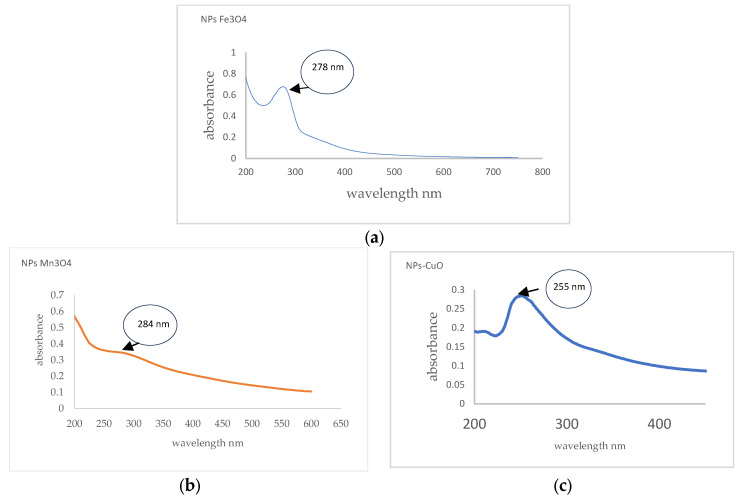
(**a**) Spectrum of UV-Vis Fe_3_O_4_ NPs, (**b**) Mn_3_O_4_ NPs, (**c**) CuO NPs.

The particles were sintered for this investigation according to the protocols described in Section 2.5. In the UV-Vis study, the Mn_3_O_4_ NPs had an absorption maximum of 284 nm, which agrees with the given data of [54]. CuO NPs are evidenced by their absorption maximum at 250 nm which is similar to that published by Prabu and Losetty (2024) [55] and Renuga et al., 2020 [56]. Fe_3_O_4_ NPs can be observed to peak in the region of 295–301 nm Fe_3_O_4_ NPs and wavelength in the band centered at 278 nm (See Figure 3a).

**Figure 4:**
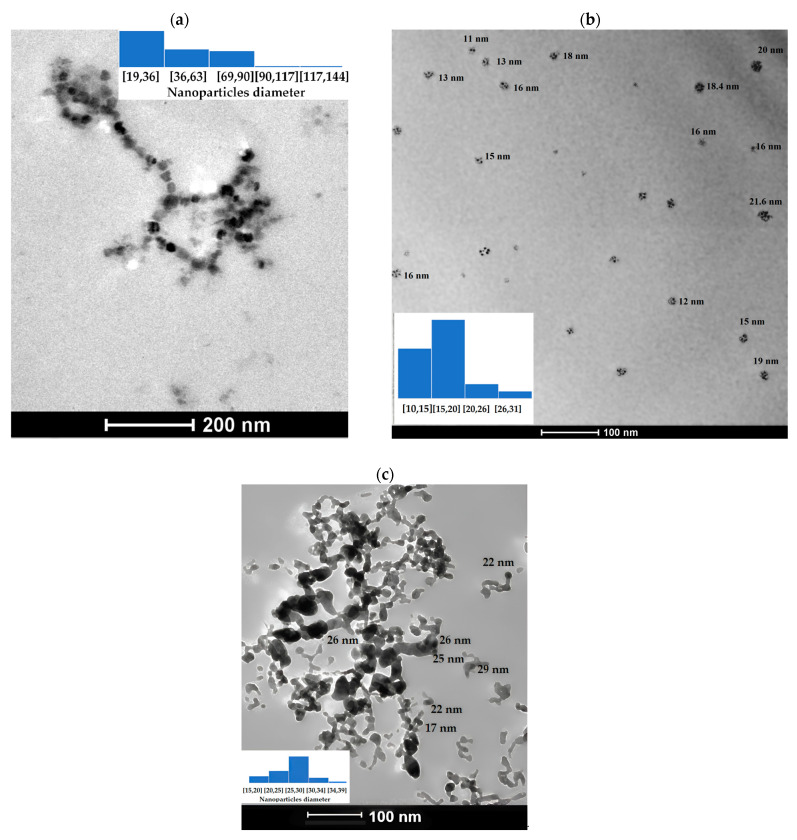
(**a**) Fe_3_O_4_ NPs, (**b**) Mn_3_O_4_ NPs, (**c**) CuO NPs.

In the TEM analyses, the “Fiji is just” program was used to process and analyze the digital images. Figure 4a shows the highest percentage is nanoparticles Fe_3_O_4_ with diameters between 9 to 36 nm while the lowest percentage of nanoparticles is about 117 to 144 nm. With respect to Mn_3_O_4_ the NPs predominant diameters are between 15 to 20 nm (Figure 4b). For CuO NPS the highest percentage of nanoparticles is found in diameters between 25 and 30 nm (Figure 4c).

**Figure 5:**
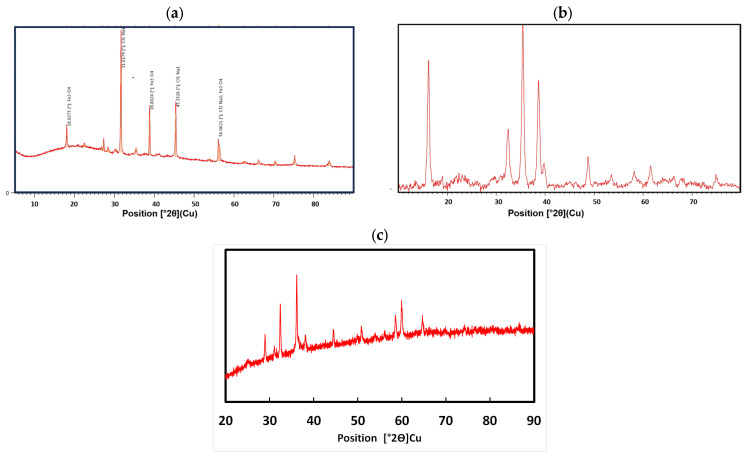
Powder X-ray diffraction (XRD) pattern of the synthesized: (**a**) NPs-Fe_3_O_4_, (**b**) NPs-Mn_3_O_4_, (**c**) NPs-CuO.

XRD studies are observed in Figure 5. The peak of the diffraction peaks of (101), (112), (200), (103), (211), (004), (220), (105), (312), (303), (321), (224), and (400) are coincident with ICDD n. No. 080-0382 and can be attributed to the tetragon in the tetragonal hausmannite phase, of the Mn_3_O_4_ spinel, which is similar to that published by [57,58,59] (Figure 5a). According to the quantification report 100% belong to tenorite (Figure 5b). The diffraction peaks (110) (002) (111) (202) (020) (202) (113) (311) (004) are indicative of a typical structure with the monoclinic topology of CuO or tenorite which is in agreement with studies by [60,61] (Figure 5c).

**Figure 6:**
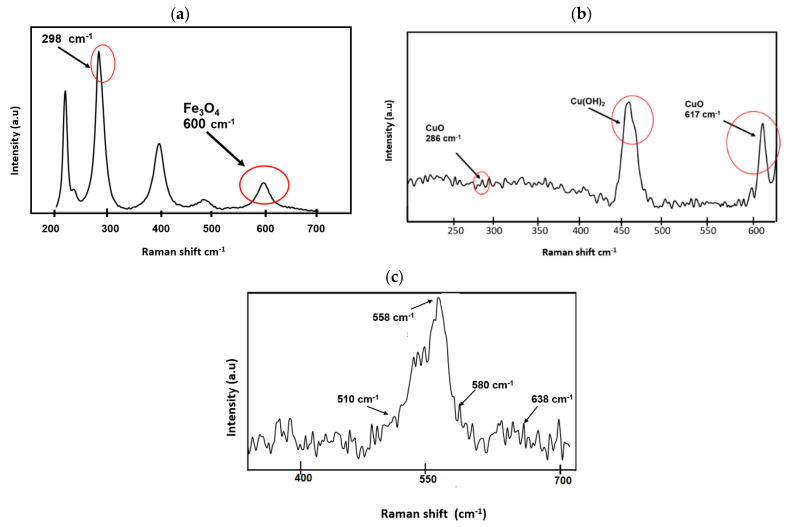
Raman spectra (**a**) CuO NPs, (**b**) Fe_3_O_4_ NPs, (**c**) manganese oxides NPs.

For NPs-Fe_3_O_4_, hematite bands are evidenced, similar to the studies of [62,63], which would indicate that oxidation to ferrous to ferric oxides occurred in the synthesis process (Figure 6a). The Raman spectrum of iron shows the peaks corresponding to the magnetite peak at 660 cm^−1^ [62,63] and 298 cm^−1^ based on [63]. Furthermore, we performed a Raman analysis. In the spectrum of the CuO nanoparticles, a band appears at 616 cm^−1^, identified as the Cu-O stretching vibration band, which coincides with the studies carried out by [62,63,64,65,66] (Figure 6b). Additionally, the characteristic peak of copper hydroxide of 500 cm^−1^ can be observed, which coincides with the research of Mayer in 1992, which has been formed in the synthesis process of copper oxides.

The Raman technique is used to analyze the local structure of manganese dioxides, especially in the case of samples with poor crystallinity [66]. Figure 6c illustrates the Raman spectrum of manganese oxide with a birnessite-type structure. The vibrational peculiarity of birnessite-type MnO_2_ is its low Raman activity. Three main characteristics can be recognized between 500–510, 575–585, and 625–650 cm^−1^. The two high wavenumber bands dominate all the spectra, while the bands in the low-frequency region appear with rather weak intensity. In the Raman spectrum of MnO_2_ (Figure 6c), three Mn-O lattice vibrations characteristic of birnessite-type MnO_2_ were observed at 510, 580, and 638 cm^−1^ [30].

**Figure 7:**
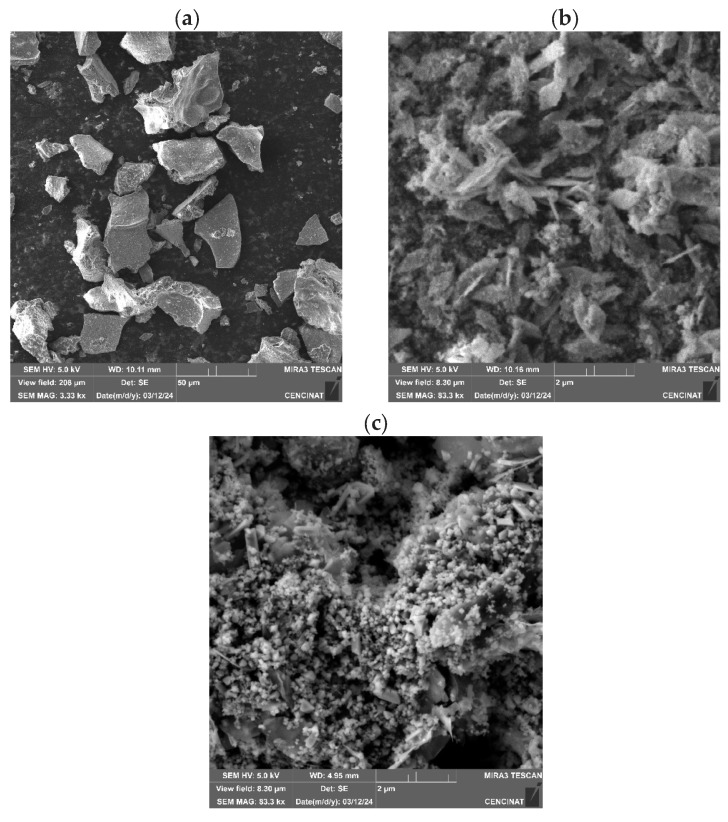
SEM images of (**a**) NPs-Fe_3_O_4_, (**b**) NPs-Mn_3_O_4_, (**c**) NPs-CuO.

Figure 7a illustrates the SEM images of NPs-Fe_3_O_4_, which indicates the crystal structure, similar to that previously described [66,67,68]. The Mn_3_O_4_ NPs are observed to be found within the organic matter of borojó, or similar to that reported by [69,70]. That is the reason why the RX diffraction of the CuO NPs, is similar to the structure of [71]. Crystalline structures are clearly visible. In the samples of magnetite, copper oxides, and manganese oxides, the surface morphology cannot be seen very clearly, because there is the borojó extract, which is the organic matter, causing the sample to exhibit low conductivity.

**Figure 8:**
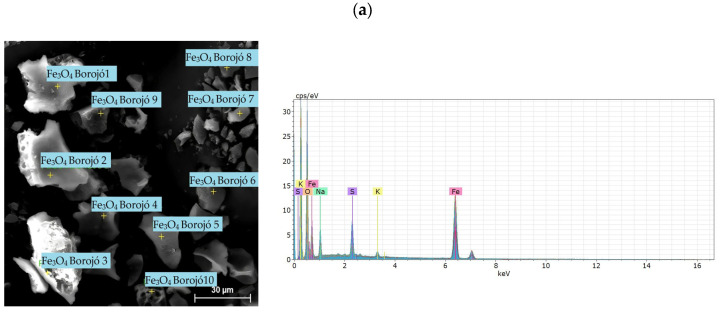

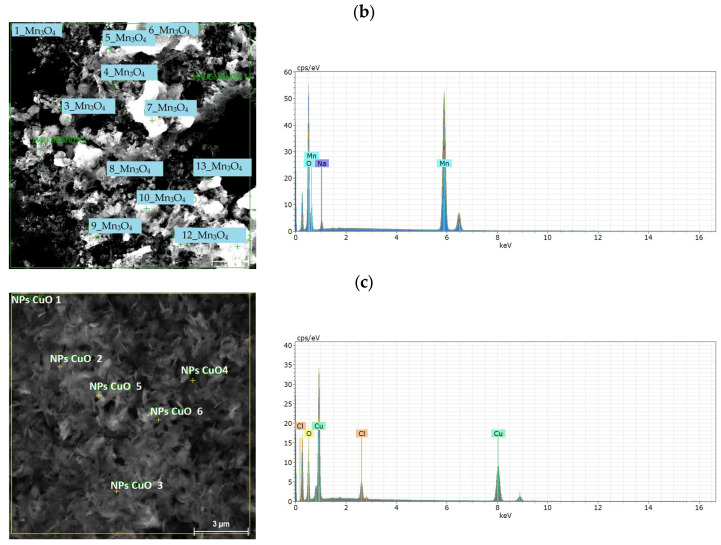
Energy dispersive X-ray spectroscopy of nanoparticles of (**a**) NPs-Fe_3_O_4_, (**b**) NPs-Mn_3_O_4_, (**c**) NPs-CuO.

In the studies of energy dispersive X-ray spectroscopy, we can observe in Figure 8a, for the NPs-Fe_3_O_4_, the presence of precursor reagents of the magnetite synthesis. For the hausmannite nanoparticles (Figure 8b) there is additionally the sodium peak whose origin is due to the alkalinization with sodium hydroxide in the synthesis process, which has not been completely eliminated in the successive washes. Finally, in Figure 8c, the chloride peak is visible, which originates from the copper chloride used in the preparation of the tenorite.

### 3.4. As Removal at the Laboratory Level

**Figure 9:**
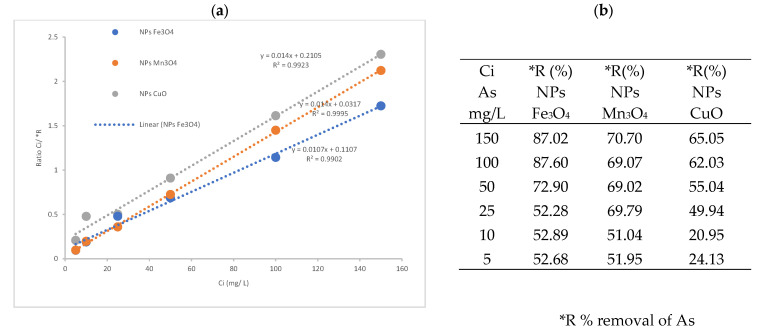
Arsenic removal: (**a**) initial and final concentrations of As using NPs-Fe_3_O_4_, NPs-Mn_3_O_4_, NPs-CuO. (**b**) Table of removal percentages.

The average removal percentages (%) of arsenic using NPs-Fe_3_O_4_ were the highest with a removal of 87.60% for initial concentration (Ci) 100 mg/L, and the least effective was with CuO-NPs, where a removal of 65.05% was achieved for Ci 150 mg/L (see Figure 9a,b).

**Figure 10:**
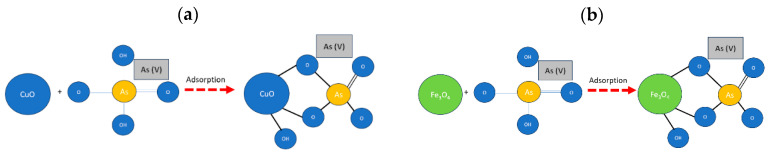
Arsenic adsorption removal process: (**a**) with CuO nanoparticles, (**b**) with Fe_3_O_4_ nanoparticles.

The removal mechanism of Fe_3_O_4_ NPs and CuO NPs with arsenic (Figure 10) is due to the effective affinity between arsenic and iron, which is due to an adsorption process [72,73], which involves chemical and physical adsorption. Additionally, the removal process may be due to the surface interaction between As(V) and ferric (hydric) oxides, which is mainly an internal sphere complexation [31,74], with respect to competing ions, sulfates, chlorides, and bicarbonates do not enter the first coordination sphere.

As removal with Mn_3_O_4_ NPs was 67.6% (at pH 2.0–8.0), monodentate and bidentate surface complexes are suggested for the adsorption of arsenic in the composite (5–16 mg/g).

Hausmannite is composed of MnO·Mn_2_O_3_ NPs, as most of these works indicate that arsenate adsorbs on the edge of MnO_2_, joining the Mn(IV) octahedra in a bidentate binuclear form [75]. More recent work has yielded that arsenate can bind to Mn(IV) octahedra in a mononuclear monodentate form and to Mn(III) octahedra in a mononuclear bidentate form.

With respect to the structure of borojó polyphenols, they enhance the stability of nanoparticles, which causes an increase in electrostatic and/or steric repulsion between particles and a decrease in van der Waals interactions and magnetic force, leading to a reduction in agglomeration and oxidation of nanoparticles [76] (Equation (1)).

(1) $$-n{Fe}^{2+}++2\mathrm{Ar}\;(\mathrm{OH})\;n\;\to \;n{Fe}^{0}+2\mathrm{nAr}=O+2nH^{+}$$

where Ar is the phenyl group and n is the number of hydroxyl groups oxidized by ${Fe}^{2+}$. The position of the hydroxyl groups on the phenolic. In addition, iron nanoparticles are highly efficient for arsenic removal, and these characteristics are based, in general, on the higher affinity of this oxyanion for active sites of the type ≡Fe–OH and ≡Fe–O–OH, which predominate on the surface of these substrates. However, due to the core-shell type structure of these nanoparticles, multiple removal processes occur, such as adsorption, absorption, precipitation, co-precipitation, oxidation, and reduction, which will predominate depending on the redox nature of the contaminant and the conditions of the medium (pH, ionic strength and redox potential) [76].

## 4. Conclusions

Nanoparticles of NPs-Mn_3_O_4_, NPs-Fe_3_O_4_, and NPs-CuO with borojum were used for arsenic removal within a water matrix.

Mn_3_O_4_ NPs, Fe_3_O_4_ NPs, and CuO NPs were synthesized by green chemistry method using borojum as an anti-caking agent and as a low-cost approach. In addition to the arsenic removal process performed by the Fe_3_O_4_, Mn_3_O_4_, and CuO nanoparticles, there is also an adsorption process by the borojum organic matter.

The properties, such as wavelength, size, distribution, morphology, and mineralogy were determined by UV-Vis, TEM, SEM, EDS, and XRD techniques, in which we could verify that the size of the NPs-Fe_3_O_4_ is between 9 nm to 46 nm, NPs-Mn_3_O_4_ average 66 nm, and NPs-CuO 5 nm to 47 nm. XRD studies confirm the mineralogy of the nanoparticles, as well as EDS and SEM studies, ratify the chemical elements present in the nanoparticles.

In addition, it was observed that the application of NPs-Mn_3_O_4_, NPs-Fe_3_O_4_, and NPs-CuO removed an average of 66% of arsenic.

Finally, it has been demonstrated that NPs could be used to remove arsenic in the waters of the Papallacta Lagoon, as part of a water treatment plant.

## Data Availability

The original contributions presented in the study are included in the article, further inquiries can be directed to the corresponding author.
